# Construction and validation of a prediction model for fall risk in hospitalized older adults with osteoporosis

**DOI:** 10.3389/fpubh.2024.1526660

**Published:** 2025-01-15

**Authors:** Li Sun, Hai-Yan Gu, Guan-Hua Xu, Jia-Wei Jiang, Ting-Ting Wang, Dan-Dan Li, Bai-Hong Cui

**Affiliations:** ^1^Department of Orthopedics, Affiliated Hospital 2 of Nantong University, Nantong, China; ^2^Department of Nursing, Affiliated Hospital 2 of Nantong University, Nantong, China; ^3^Department of Spinal Surgery, Affiliated Hospital 2 of Nantong University, Nantong, China; ^4^Department of Endocrine, Affiliated Hospital 2 of Nantong University, Nantong, China; ^5^Department of Intervention, Affiliated Hospital 2 of Nantong University, Nantong, China

**Keywords:** fall, nomogram, nursing, osteoporosis (OP), prediction model, risk factors

## Abstract

**Objective:**

The aim of this study is to develop and validate a prediction model for fall risk factors in hospitalized older adults with osteoporosis.

**Methods:**

A total of 615 older adults with osteoporosis hospitalized at a tertiary (grade 3A) hospital in Nantong City, Jiangsu Province, China, between September 2022 and August 2023 were selected for the study using convenience sampling. Fall risk factors were identified using univariate and logistic regression analyses, and a predictive risk model was constructed and visualized through a nomogram. Model performance was evaluated using the area under the receiver operator characteristic curve (AUC), Hosmer-Lemeshow goodness-of-fit test, and clinical decision curve analysis, assessing the discrimination ability, calibration, and clinical utility of the model.

**Results:**

Based on logistic regression analysis, we identified several significant fall risk factors for older adults with osteoporosis: gender of the study participant, bone mineral density, serum calcium levels, history of falls, fear of falling, use of walking aids, and impaired balance. The AUC was 0.798 (95% CI: 0.763–0.830), with a sensitivity of 80.6%, a specificity of 67.9%, a maximum Youden index of 0.485, and a critical threshold of 121.97 points. The Hosmer-Lemeshow test yielded a χ^2^ value of 8.147 and *p* = 0.419, indicating good model calibration. Internal validation showed a C-index of 0.799 (95% CI: 0.768–0.801), indicating the model’s high discrimination ability. Calibration curves showed good agreement between predicted and observed values, confirming good calibration. The clinical decision curve analysis further supported the model’s clinical utility.

**Conclusion:**

The prediction model constructed and verified in this study was to predict fall risk for hospitalized older adults with osteoporosis, providing a valuable tool for clinicians to implement targeted interventions for patients with high fall risks.

## 1 Introduction

Osteoporosis (OP) is a systemic bone disease associated with aging (1). With rapid population aging, OP has become the fourth most prevalent chronic disease among urban residents in China, with its incidence increasing annually (2). As reported from large-sample studies, the prevalence of OP among older adults in China in recent years ranges between 36 and 37.7%, highlighting its growing significance as an important public health concern in the country (3). Due to the often subtle nature of its symptoms and relatively low diagnosis rate, individuals with OP often present with osteoporotic fractures (OPF) as the initial symptom and primary reason for seeking medical attention. As the most severe complication of OP, OPF is also the predominant type of fracture among older adults (4).

A fall is defined as an incident involving a sudden, involuntary, and unintentional change in an individual’s posture, resulting in a descent to the ground or a lower surface. Falls are an independent risk factor for OPF (5). Falls pose a significant challenge for independent living among older adults with OP as they are associated with several negative consequences such as disability, fear of falling (FoF) again, compromised quality of life, fractures, and even death. Falls also contribute to increased healthcare burdens. Given these serious repercussions, the evaluation of fall risk in older adults with OP and implementing preventive measures assumes much significance.

A risk prediction model provides a scientific and precise tool to assess fall risk in older adults, thus enabling the implementation of targeted preventive interventions to effectively lower the incidence of falls. The focus of current research on falls in individuals with OP is mainly on risk factors, with very few studies addressing the development of prediction models for fall risks in this group.

In this study, a fall risk prediction model was constructed by investigating the risk factors for falls in older adults with OP so as to provide a reference for the healthcare professionals to identify individuals at high risk of falls and implement preventative strategies to reduce falls.

## 2 Study sample and methods

### 2.1 Study participants and sample size estimation

Older adults with OP who were hospitalized in the departments of spine surgery, endocrinology, and geriatrics of a Grade 3A hospital in Nantong City, Jiangsu province, China, between September 2022 and August 2023, were selected for the study through convenience sampling.

The criteria for inclusion were as follows: (1) patients whose diagnosis conformed to the criteria for OP in the *Guidelines for Diagnosis and Treatment of Primary Osteoporosis* (6), specifically, a bone mineral density (BMD) T-score of ≤ −2.5 SD, as measured by dual-energy X-ray absorptiometry (DXA); (2) individuals aged 61–78 years categorized as older adults according to the new classification of global life expectancy of the World Health Organization (WHO)^1^; (3) patients who were conscious and alert, and able to engage in verbal communication; and (4) patients who signed the informed consent.

Exclusion criteria were as follows: (1) patients who were bedridden for a long time; (2) those with severe organ diseases or organ failure who were unable to cooperate with the study procedures; and (3) patients who are blind, deaf, or have very severe hearing loss (mean hearing threshold ≥81dBHL) (7).

Following were the drop-out criteria: patients who could not complete the follow-up visits due to death or other causes.

Sample size estimation: As per criteria for using logistic regression analysis to study impact factors of relevant variables, the sample size should be 15–20 times the number of variables (8). Additionally, based on a literature review for this study, qualitative interviews, and expert consultations via letter correspondence, 37 preliminary risk factors were identified, suggesting a required sample size of 555–740 participants. Accounting for a 10% loss to follow-up, the sample size range was adjusted to 610–814 participants. A total of 615 individuals were finally enrolled.

This study was approved by the Ethics Committee of the Second Affiliated Hospital of Nantong University (approval no. 2022YKY034).

### 2.2 Study methods

#### 2.2.1 Study tools

##### 2.2.1.1 General data

Study participant demographics, such as sex, age, marital status, education level, living conditions, and caregiving arrangements, among other aspects, were collected using a questionnaire.

##### 2.2.1.2 Biochemical indicators

An automatic biochemical analyzer (Type 7,600, Hitachi) was used to analyze biochemical parameters such as serum albumin, serum calcium, total procollagen type I N-terminal propeptide (TP1NP), *β* C-terminal cross-linked telopeptides of type I collagen (β-CTX), and parathyroid hormone (PTH). Blood samples from the elbow veins were collected by ward nurses from each patient after overnight fasting for 8 h. All tests were conducted by professional personnel of the medical laboratory department of the hospital.

##### 2.2.1.3 BMD measurement

BMD was measured using the US Hologic Discovery Wi (S/N 86856) device using the DXA method. Procedures for BMD measurement included the following: (1) patients were required to change into the hospital gown before undergoing the examination; (2) basic information of the patients, such as name, age, admission number, height, and body weight, was recorded; (3) patients were in a supine position on the instrument during the measurement; and (4) all BMD measurements were performed by professional staff from the medical laboratory department.

##### 2.2.1.4 Drug classification

The Anatomical Therapeutic Chemical (ATC) classification system (9), officially endorsed by the WHO was used to code the types of medications the patients were being administered.

##### 2.2.1.5 Mobility assessment

Mobility was evaluated using the 3-meter Timed Up and Go Test (TUGT) (10). The patients were seated in a 45 cm-high straight back chair with both hands placed on the armrests. A marker was placed at a distance of three meters from the chair. When the evaluator gave the “Start” command, the patient was instructed to stand up and walk to the marker, turn around, and return to sit down in the chair. The time from getting up to sitting back down after returning was recorded with a stopwatch. The test was performed independently by the patient without any external assistance. A TUGT time of >15 s indicated decreased mobility.

##### 2.2.1.6 Muscular strength evaluation

Upper limb muscular strength was evaluated using grip strength. The patients used their dominant hand to grip a spring-type dynamometer to perform a maximum isometric contraction. The test was repeated twice, and the higher reading was recorded. A grip strength of <28.0 kg in males and < 18.0 kg in females indicated decreased grip strength (11).

Lower limb muscular strength was evaluated using the Five Times Sit-to-Stand Test (FTSST) (12), using the same equipment as the TUGT. The patients were seated in a chair with the chair back against a wall, arms crossed over the chest, back straight away from the chair back, and feet flat on the ground. After the evaluator gave the “Start” command, the patients had to stand up from the chair fully and then sit down completely, repeating the action five consecutive times as quickly as possible. The total time from start from standing up initially to the final complete sit was recorded with a stopwatch. The patients completed the whole test independently without any external assistance. FTSST cutoff values of >11 s (ending with sitting position) and > 10 s (ending with standing position) were considered as decreased lower limb muscle strength (13).

##### 2.2.1.7 Balance assessment

The Berg Balance Scale (BBS) (14) was used to evaluate the participants’ ability to balance. This scale consists of 14 test items, each with five response options scored from 0 to 4. The total score ranges from 0 to 56, and a score < 40 indicates the need for a walking aid and reduced balance ability.

##### 2.2.1.8 Cognitive status

The Clock Drawing Test (CDT) (15) was used for a quick assessment of the cognitive functioning status of the participants in the study. This method is both simple as well as practical and is applicable to a wide range of populations. The test consists of the following four key steps: drawing a complete circle, correctly placing the 12 numbers on the clock face, positioning the numbers accurately, and setting the clock hands correctly. Each correctly completed step is scored 1 point, and the total score is 4. A score of ≤2 indicates cognitive impairment.

##### 2.2.1.9 Fear of falling

Fear of falling was assessed using a single-item questioning method, i.e., the patients were asked, “Do you worry about or fear falling?” Responses were categorized as “no” indicating no fear of falling, while “a little” or “very” indicated a fear of falling.

##### 2.2.1.10 Screening for depression

The short form of the Geriatric Depression Scale (GDS-5) (16) was used to evaluate symptoms of depression. This scale consists of five items, each answered with “yes” or “no” by the respondent as per their own condition, with a score of 1 or 0, respectively. Negative items are reverse-scored. The total score ranges from 0 to 5, and a score ≥ 2 indicates the presence of depressive symptoms.

##### 2.2.1.11 Sleep experience

The patients’ average weekly exercise intensity and time were measured and classified according to Sports Prescription of Chinese Expert Consensus (17). No: No exercise at any time; Light: Less than 150 min of moderate-intensity exercise or less than 75 min of vigorous-intensity physical activity per week; Moderate: 150 min or more of moderate-intensity exercise but less than 300 min per week or 75 min of vigorous-intensity exercise but less than 150 min per week; A lot: At least 300 min of moderate-intensity exercise or 150 min of vigorous-intensity exercise per week.

##### 2.2.1.12 Screening for depression

Sleep Quality was assessed using the Pittsburgh Sleep Quality Index (PSQI). The PSQI was developed by Buysse et al. (18) and is a self-report assessment tool to evaluate sleep quality over one-month period. A global score and seven component scores can be derived from the scale. The component scores are the following: subjective sleep quality, sleep latency, sleep duration, sleep efficiency, sleep disturbances, sleeping medications and daytime dysfunction. Each component is scored on a scale from 0 to 3, with the total scores ranging from 0–21; where a higher score describes poorer sleep quality.

##### 2.2.1.13 Hearing test and visual acuity assessment

Patients wear headphones in both ears in a closed audiometry room in the ent outpatient department, and the hearing test is made by the pure tone issued by the ent doctor in the control room. If you hear the sound, press the button, mark it, and draw a listening curve. The average listening threshold of the four frequencies of 500 Hz, 1,000 Hz, 2000 Hz and 4,000 Hz is taken as the average listening threshold. Normal hearing means that the average hearing threshold is ≤25 dBHL, and decreased hearing means that the average hearing threshold is between 60 and 80 dBHL.

Visual acuity test was performed by an ophthalmologist and Snellen visual acuity chart was used to screen older adult patients for visual impairment. The older adult are advised to wear glasses (if equipped) for Snellen visual acuity chart examination. The corrected visual acuity of 0.8 and above is normal visual acuity, and the corrected visual acuity of less than 0.8 is decreased visual acuity. If the corrected visual acuity of the two eyes is different, the better visual acuity shall be taken as the standard.

#### 2.2.2 Safety guarantee

During the evaluation process, the respondents were required to be accompanied by the evaluator, who maintained a distance of no more than 1 meter from them. This proximity allowed the evaluator to provide immediate preventive and protective measures to avoid accidents such as hospital falls during the evaluation process. The patients were required to wear a finger pulse oximeter during the entire process. The evaluation was discontinued under the following circumstances and resumed only once the symptoms improved: (1) The heart rate exceeded the target heart rate; target heart rate was calculated as = (220 – age – resting heart rate) × 50% + resting heart rate; and (2) SPO_2_ levels dropped to <88%, and the patient experienced noticeable discomfort.

#### 2.2.3 Data collection and quality control methods

A research group was formed for the study, consisting of two nursing postgraduates and four specialist nurses with intermediate or higher qualifications from the departments of orthopedics, endocrinology, and geriatrics. With approval from the heads of the departments, the team members collected data on relevant risk factors using a structured risk factor questionnaire from patients admitted in various wards across the spine surgery, endocrinology, and geriatrics units.

Post-discharge from the hospital, study participants were followed up via telephone calls by the team to document any occurrence of falls and their specific circumstances. The team made telephonic follow-ups once every 3 months, for a total of two follow-ups. Study participants who experienced a fall(s) within 6 months were categorized into the Fall group, while those without falls were included in the Non-fall group.

Prior to the survey, the investigators were trained on filling out the questionnaires and risk evaluation so as to ensure consistency across the study. The investigators followed the criteria for inclusion and exclusion when selecting participants for the study, explaining the objectives and procedures involved in the study. The questionnaires were filled out after the participants gave their consent. Contact details of the participants were collected, and follow-up appointments were scheduled in advance to inquire about fall incidents.

### 2.3 Statistical analysis

Data collection, verification, and input were performed by two individuals. Statistical analysis was conducted using SPSS 27.0. This study calculated the required sample size based on Logistic regression analysis, and the required sample size for each variable was 5–10. A total of 37 risk factors were obtained through literature research, qualitative interviews and expert correspondence. According to the incidence of falls in older adult osteoporosis patients was 14.6%. Considering the possibility of lost follow-up of some research subjects, the incidence was set as 20%, and it was calculated that the sample size required for this model construction was at least 37 × 5÷0.146÷0.8 = 1,584 cases, and the sample size of the verification set was 1/4 ~ 1/2 of the modeling set. Therefore, it is calculated that the sample size required to verify the model is 1,584× (1/4 ~ 1/2) = 396 ~ 792 cases. The final sample size of this study was determined as 615 cases.

Continuous variables conforming to a normal distribution were represented using the mean ± standard deviation (X̄ ± S), and the t*-*test was used for inter-group comparisons. For continuous variables not in a normal distribution, data were expressed as the median and quartile [M (P25, P75)], and the rank-sum test was used for inter-group comparisons. Categorical data were described as frequencies and percentages (%), and the Chi-square χ^2^ test or Fisher’s exact probability method was used for inter-group comparisons.

In univariate analysis, independent variables with statistical significance (*p* < 0.05) were included in a logistic regression analysis. The variance inflation factor (VIF) was used in collinearity diagnosis to assess multi-collinearity, with VIF > 10 indicating collinearity. A predictive equation was then constructed based on the partial regression coefficients of various variables, and the prediction model for fall risk in older adults with OP was created. R4.3.0 software was used to construct the nomogram, and internal validation of the model was done using the Bootstrap self-sampling method.

The receiver operator characteristic (ROC) curve was used to calculate the area under curve (AUC) to evaluate the model’s discriminatory ability. Hosmer-Lemeshow test was used to evaluate the calibration degree of the Logistic regression model, and *p* value >0.05 indicated that the calibration degree of the model was good. While clinical decision curve analysis (DCA) was utilized to evaluate the model’s clinical effectiveness. A significance level of *α* = 0.05 was set for the tests.

## 3 Results

### 3.1 General data and fall occurrences of the study objects

A total of 615 older adults with OP were included in the study. Among them, 580 (94.31%) completed the follow-up, while 35 participants (5.69%) were lost to follow-up and subsequently excluded from the risk analysis and model construction.

Among the 580 participants who completed the follow-up, there were 131 males (22.59%) and 449 females (77.41%). They were aged 61–78 years, with a mean age of (69.70 ± 4.51) years. In terms of their level of education, 82 participants (14.14%) were illiterate, 324 (55.86%) had completed junior high school or lesser, and 174 participants (30%) had completed senior high school or above. With respect to their living arrangements, there were 60 participants (10.34%) who lived alone, 268 (46.21%) lived with their children, and 252 (43.45%) lived with their spouses.

Based on fall occurrence within 6 months of discharge from the hospital, there were 71 participants (12.24%) in the Fall group and 509 (87.76%) in the Non-fall group. In the Fall group, 12 participants had a total of 23 instances of falls, and 29 participants reported 49 injurious falls.

Regarding the time of occurrence, 85% of the falls occurred during the daytime. As for the places, 59% of falls occurred outdoors, and 41% of falls occurred indoors. Regarding the direct causes of falls, 31% of the falls were due to body discomfort experienced by the participants. Environmental factors accounted for 41% of falls, and these causes included obstacles on the ground and wet or uneven road surfaces, among others. A total of 12% of falls were due to high-risk activities of participants, such as riding a bicycle, rushing to beat traffic lights, and being distracted.

### 3.2 Univariate analysis of fall risk factors in hospitalized older adults with osteoporosis

Univariate analysis was performed using various risk factors as the independent variable, and the occurrence of falls served as the dependent variable. The analysis revealed significant differences between the Fall and Non-fall groups (*p* < 0.05) in a total of 14 variables, i.e., gender, exercise habits, history of falls, fear of falling, depressive symptoms, decreased balance ability, gait, use of walking aids, muscular strength, BMD, serum calcium levels, serum 25-hydroxyvitamin D levels, as well as a history of complicated cerebral stroke and Parkinson’s disease. The results are detailed in Table 1.

**Table 1:** Univariate analysis of fall risk factors among older adults with osteoporosis.

Risk factor	Number of cases (*n* = 580)	Non−fall group (*n* = 509)	Fall group (*n* = 71)	*p* value
Age (years)	69.70 ± 4.51	69.60 ± 4.58	70.42 ± 4.01	0.149
Sex				0.033*
Male	131 (22.59)	122 (23.97)	9 (12.68)	
Female	449 (77.41)	387 (76.03)	62 (87.32)	
BMI (kg/m^2^)				0.316
<18.5	23 (3.97)	21 (4.13)	2 (2.82)	
18.5–23.9	307 (52.93)	268 (52.65)	39 (54.93)	
24.0–27.9	198 (34.14)	178 (34.97)	20 (28.17)	
Education level				0.087
Illiterate	82 (14.14)	39 (7.66)	43 (60.56)	
Junior high school or below	324 (55.86)	301 (59.14)	23 (32.39)	
Senior high school or above	174 (30.00)	169 (33.20)	5 (7.04)	
Living arrangement				0.008*
Living alone	60 (10.34)	46 (9.04)	14 (19.72)	
With children	268 (46.21)	244 (47.94)	24 (33.80)	
With spouse	252 (43.45)	219 (43.03)	33 (46.48)	
Exercise habit				<0.001*
No	189 (32.59)	152 (29.86)	37 (52.11)	
Light	120 (20.69)	101 (19.84)	19 (26.76)	
Moderate	211 (36.38)	198 (38.90)	13 (18.31)	
A lot	60 (10.34)	58 (11.39)	2 (2.82)	
Sleep experience				0.212
Very good	13 (2.24)	13 (2.55)	0 (0.00)	
Relatively good	486 (83.79)	428 (84.09)	58 (81.69)	
Relatively poor	75 (12.93)	62 (12.18)	13 (18.31)	
Very poor	6 (1.03)	6 (1.18)	0 (0.00)	
Increased nocturia (≥3 times/night)				0.057
No	550 (94.83)	486 (95.48)	64 (90.14)	
Yes	30 (5.17)	23 (4.52)	7 (9.86)	
History of falls (within last one year)				0.03*
No	362 (62.41)	326 (64.05)	36 (50.70)	
Yes	218 (37.59)	183 (35.95)	35 (49.30)	
History of fracture				0.637
No	206 (35.52)	179 (35.17)	27 (38.03)	
Yes	374 (64.48)	330 (64.83)	44 (61.97)	
History of vertigo				0.162
No	518 (89.31)	458 (89.98)	60 (84.51)	
Yes	62 (10.69)	51 (10.02)	11 (15.49)	
Cognitive impairment				0.623
No	501 (86.38)	441 (86.64)	60 (84.51)	
Yes	79 (13.62)	68 (13.36)	11 (15.49)	
Fear of falling				<0.001*
No	223 (38.45)	211 (41.45)	12 (16.90)	
Yes	357 (61.55)	298 (58.55)	59 (83.10)	
Depressive symptoms				0.02*
No	559 (96.38)	494 (97.05)	65 (91.55)	
Yes	21 (3.62)	15 (2.95)	6 (8.45)	
Unusual sensations in lower limbs				0.066
No	512 (88.28)	454 (89.19)	58 (81.69)	
Yes	68 (11.72)	55 (10.81)	13 (18.31)	
Mobility				0.899
Normal	43 (7.41)	38 (88.37)	5 (11.63)	
Decreased	537 (92.59)	471 (92.53)	66 (92.96)	
Balance ability				<0.001*
Normal	531 (91.55)	481 (94.50)	50 (70.42)	
Decreased	49 (8.45)	28 (5.50)	21 (29.58)	
Gait				0.043*
Steady	515 (88.79)	457 (89.78)	58 (81.69)	
Unsteady	65 (11.21)	52 (10.22)	13 (18.31)	
Use of walking aids				0.046*
No	463 (79.83)	400 (78.59)	63 (88.73)	
Yes	117 (20.17)	109 (21.41)	8 (11.27)	
Grip strength				0.035*
Normal	383 (66.03)	344 (67.58)	39 (54.93)	
Decreased	197 (33.97)	165 (32.42)	32 (45.07)	
Lower limb muscle strength				0.002*
Normal	209 (36.03)	195 (38.31)	14 (19.72)	
Decreased	371 (63.97)	314 (61.69)	57 (80.28)	
Hearing				0.105
Normal	547 (94.31)	483 (94.89)	64 (90.14)	
Decreased	33 (5.69)	26 (5.11)	7 (9.86)	
Vision				0.126
Normal	546 (94.14)	482 (94.70)	64 (90.14)	
Decreased	34 (5.86)	27 (5.30)	7 (9.86)	
BMD T−score	−3.10 (−2.30, −3.60)	−3.00 (−2.20, −3.60)	−3.30 (−2.70, −3.85)	0.012*
Alb (g/L)	36.91±4.84	37.01±4.97	36.17±3.67	0.169
Serum calcium (mmol/L)	2.18 (2.09, 2.26)	2.18 (2.10, 2.27)	2.15 (2.06, 2.22)	0.024*
TP1NP (g/L)	60.59 (43.81, 77.21)	60.42 (43.89, 77.10)	62.34 (42.45, 82.53)	0.956
β−CTX (ng/mL)	0.69 (0.41, 1.06)	0.70 (0.41, 1.06)	0.62 (0.39, 1.10)	0.975
PTH (pg/mL)	53.35 (36.38, 74.50)	53.30 (37.20, 74.90)	55.30 (33.05, 70.90)	0.404
25 (OH)V (ng/mL)	18.75 (15.75, 24.07)	18.90 (15.98, 24.43)	17.69 (14.47, 20.98)	0.029*
Cerebral stroke				0.033*
No	535 (92.24)	474 (93.12)	61 (85.92)	
Yes	45 (7.76)	35 (6.88)	10 (14.08)	
Hypertension				0.139
No	349 (60.17)	312 (61.30)	37 (52.11)	
Yes	231 (39.83)	197 (38.70)	34 (47.89)	
Diabetes				0.066
No	464 (80.00)	413 (81.14)	51 (71.83)	
Yes	116 (20.00)	96 (18.86)	20 (28.17)	
Heart disease				0.201
No	516 (88.97)	456 (89.59)	60 (84.51)	
Yes	64 (11.03)	53 (10.41)	11 (15.49)	
Parkinson’s disease				0.028*
No	558 (96.21)	493 (96.86)	65 (91.55)	
Yes	22 (3.79)	16 (3.14)	6 (8.45)	
Arthritis				0.386
No	558 (96.21)	491 (96.46)	67 (94.37)	
Yes	22 (3.79)	18 (3.54)	4 (5.63)	
Hyperosteogeny				0.552
No	449 (77.41)	396 (77.80)	53 (74.65)	
Yes	131 (22.59)	113 (22.20)	18 (25.35)	
Other complications				0.15
No	498 (85.86)	441 (86.64)	57 (80.28)	
Yes	82 (14.14)	68 (13.36)	14 (19.72)	
Medication				0.113
No	288 (49.66)	259 (50.88)	29 (40.85)	
Yes	292 (50.34)	250 (49.12)	42 (59.15)	
Continuous medication ≥ 3months				0.468
No	293 (50.52)	260 (51.08)	33 (46.48)	
Yes	287 (49.48)	249 (48.92)	38 (53.52)	
Drug type				0.373
No	304 (52.41)	266 (52.26)	38 (53.52)	
1–3 types	196 (33.79)	176 (34.58)	20 (28.17)	
≥3 types	80 (13.79)	67 (13.16)	13 (18.31)	
Class A drugs^#^				0.053
No	452 (77.93)	403 (79.17)	49 (69.01)	
Yes	128 (22.07)	106 (20.83)	22 (30.99)	
Class B drugs^#^				0.353
No	565 (97.41)	497 (97.64)	68 (95.77)	
Yes	15 (2.59)	12 (2.36)	3 (4.23)	
Class C drugs^#^				0.115
No	344 (59.31)	308 (60.51)	36 (50.70)	
Yes	236 (40.69)	201 (39.49)	35 (49.30)	
Class D drugs^#^				0.709
No	579 (99.83)	508 (99.80)	71 (100.00)	
Yes	1 (0.17)	1 (0.20)	0 (0.00)	
Class H drugs^#^				0.402
No	575 (99.14)	504 (99.02)	71 (100.00)	
Yes	5 (0.86)	5 (0.98)	0 (0.00)	
Class J drugs^#^				0.709
No	579 (99.83)	508 (99.80)	71 (100.00)	
Yes	1 (0.17)	1 (0.20)	0 (0.00)	
Class L drugs^#^				0.264
No	577 (99.48)	507 (99.61)	70 (98.59)	
Yes	3 (0.52)	2 (0.39)	1 (1.41)	
Class M drugs^#^				0.982
No	572 (98.62)	502 (98.62)	70 (98.59)	
Yes	8 (1.38)	7 (1.38)	1 (1.41)	
Class N drugs^#^				0.114
No	564 (97.24)	497 (97.64)	67 (94.37)	
Yes	16 (2.76)	12 (2.36)	4 (5.63)	
Class V drugs^#^				0.727
No	567 (97.76)	498 (97.84)	69 (97.18)	
Yes	13 (2.24)	11 (2.16)	2 (2.82)	

BMI, body mass index; PTH, parathyroid hormone; β-CTX, β C-terminal cross-linked telopeptides of type I collagen; TP1NP, Total procollagen type I N-terminal propeptide; 25(OH)D, 25-hydroxy-vitamin D; *p < 0.05; #Drug class codes (A: Digestive tract and metabolism; B: Blood and blood forming organs; C: Cardiovascular system; D: Dermatological drugs, G: Genitourinary system and sex hormones; H: Non-sex hormones and insulin hormone system drugs; J: Systemic anti-infective drugs; L: Anti-tumor agents and immunomodulators; M: Musculoskeletal system drugs; N: Nervous system drugs; V: Miscellaneous drugs).

### 3.3 Logistic regression analysis of fall risks in hospitalized older adults with OP

A logistic regression analysis was conducted using the 14 variables from the univariate analysis that had a *p* value <0.05 as independent variables, and the occurrence of fall was the dependent variable. The method of assigning values for the variables is shown in Table 2. Seven factors, namely, gender, BMD, serum calcium levels, history of falls, fear of falling, use of walking aids, and decreased balance ability, were included in the regression model. Risk factors for falls in older adults with OP included being female, a history of falls, fear of falling, and decreased balance ability, while higher BMD, higher serum calcium levels, and use of walking aids were the protective factors among older adults with OP. The results are presented in Table 3.

**Table 2:** Values assigned to independent variables.

Variable name	Value assigned
Gender	Male = 1; Female = 2
BMD T-score	Continuous variable
Serum calcium	Continuous variable
Fall history	No = 0; Yes = 1
Fear of falling	No = 0; Yes = 1
Balance ability	Good = 1; Poor = 2
Walking aid	No = 0; Yes = 1

**Table 3:** Logistic regression analysis of fall risk factors in older adults with osteoporosis.

Variable	*β*	*SE*	*Wald*	*p*	*OR*	*95%CI*
Gender: female	0.998	0.414	5.815	0.016	2.712	1.205–6.103
BMD T-score	−0.338	0.164	4.264	0.039	0.713	0.518–0.983
Serum calcium levels	−3.246	0.939	11.938	<0.001	0.039	0.006–0.245
History of falls	0.646	0.288	5.028	0.025	1.907	1.085–3.353
Fear of falling	0.994	0.349	8.094	0.004	2.701	1.362–5.356
Decreased balance ability	2.29	0.446	26.311	<0.001	9.878	4.117–23.698
Use of walking aids	−1.57	0.521	9.088	0.003	0.208	0.075–0.577
Constant	0.078	2.275	0.001	0.973	1.081	

### 3.4 Construction of the prediction model for fall risk in hospitalized older adults with OP

The following logistic regression equation was constructed based on the identified risk factors: Logit(*P*) = 0.078 + 0.998 × females −0.338 × T-score – 3.246 × serum calcium +0.646 × fall history +0.994 × fear of falling +2.29 × decreased balance ability −1.57 × walking aid.

**Figure 1:**
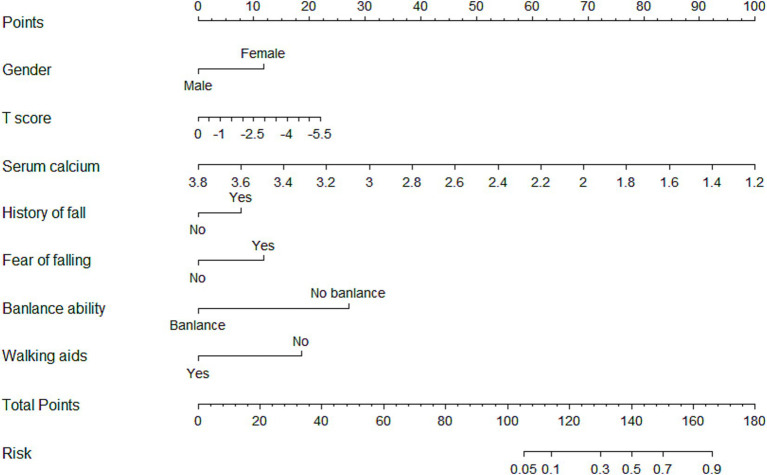
Nomogram of fall risk factors among older adults with osteoporosis.

Using R4.3.0 software, a nomogram was created to visually represent the prediction model (Figure 1). The regression coefficients for each factor were used to assign scores. Each variable was aligned with a vertical score scale, allowing for individual factor scores to be determined. The total score, which was the sum of the individual scores, was then converted into a fall probability through a functional relationship.

### 3.5 Evaluation and verification of the prediction model for fall risk in hospitalized older adults with OP

**Figure 2:**
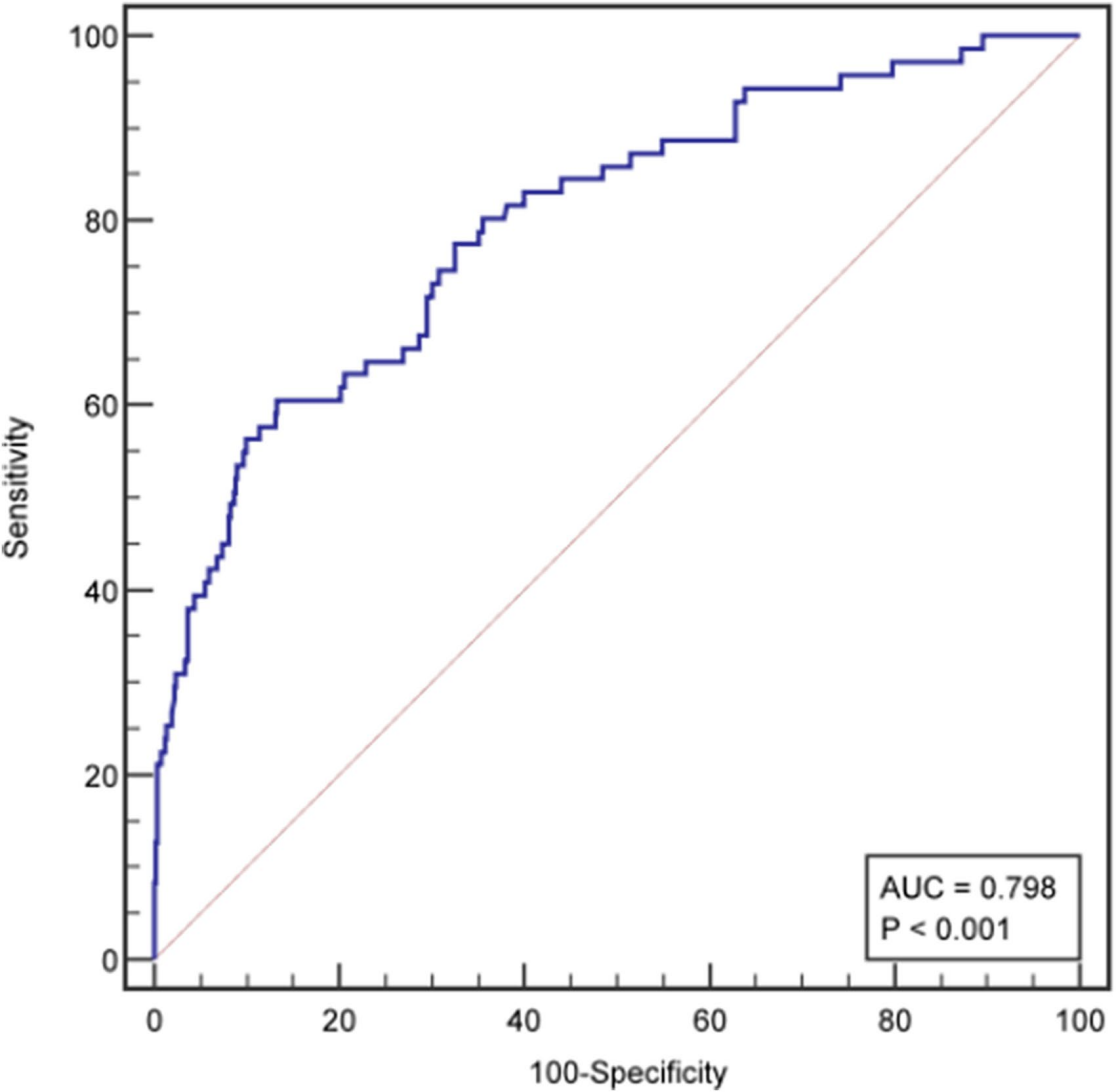
ROC curve of the fall risk prediction model for older adults with osteoporosis.

**Figure 3:**
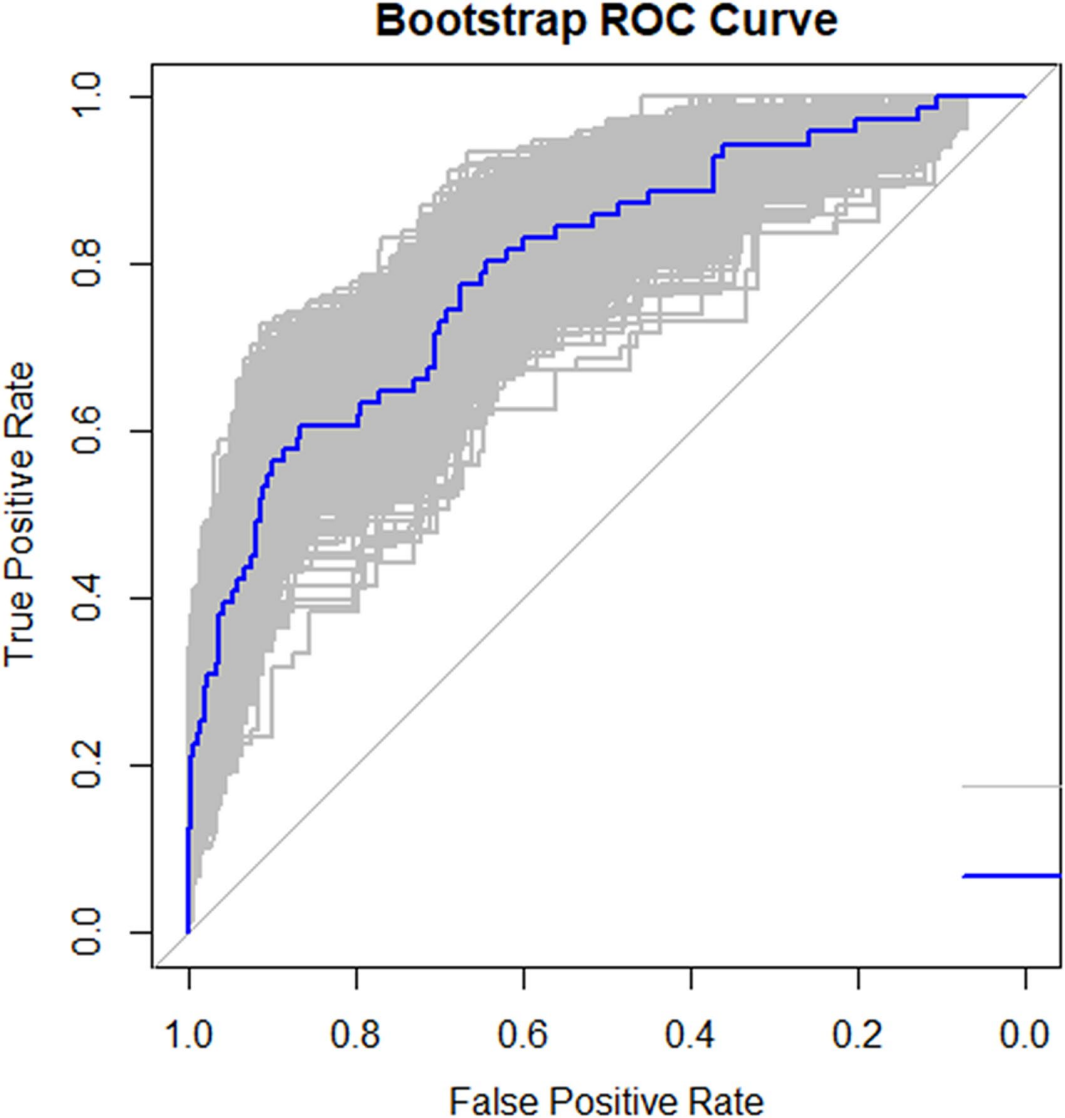
Internal validation of the fall risk prediction model for older adults with osteoporosis.

**Figure 4:**
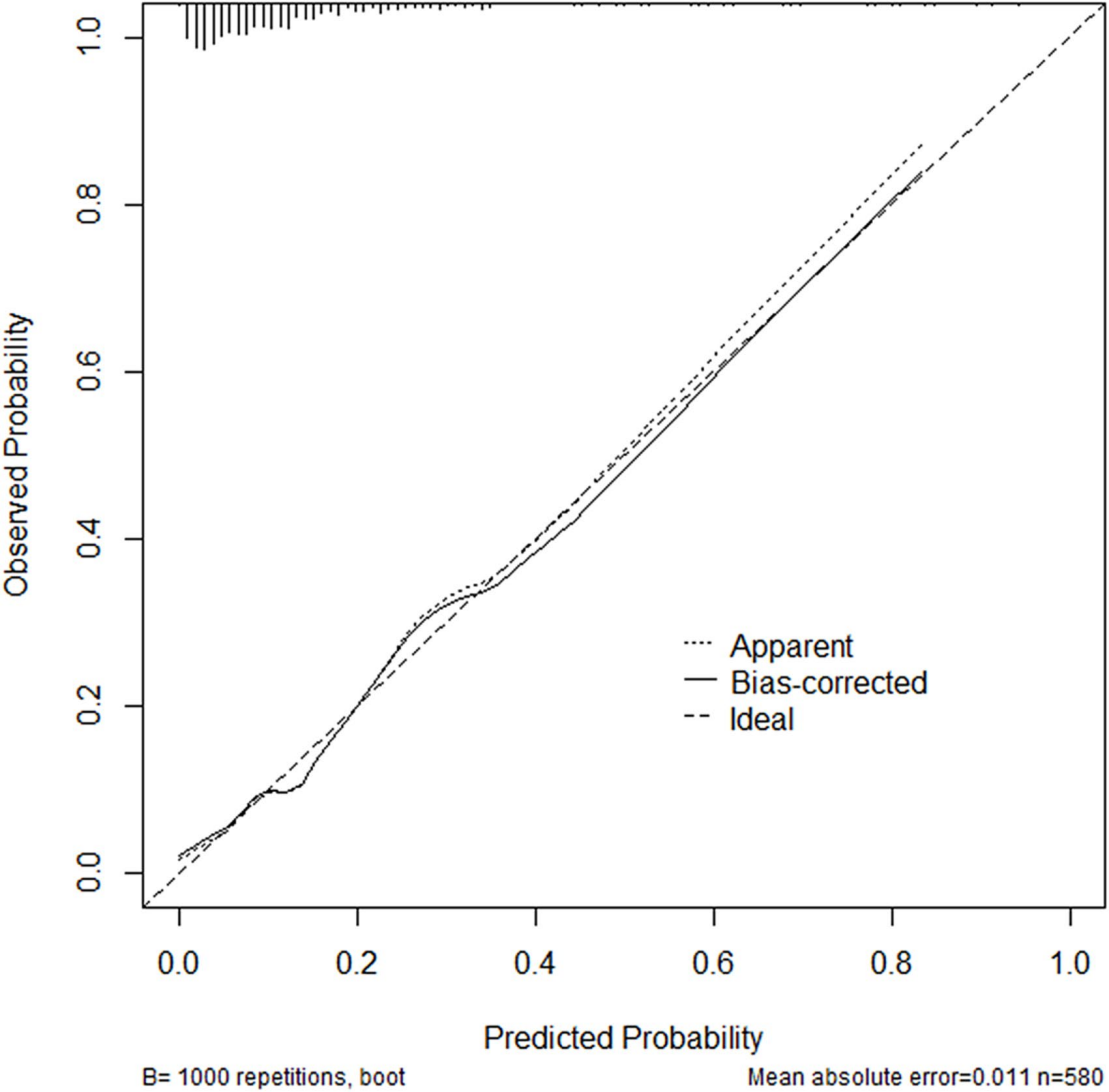
Calibration curve diagram of the fall risk prediction for older adults with osteoporosis.

**Figure 5:**
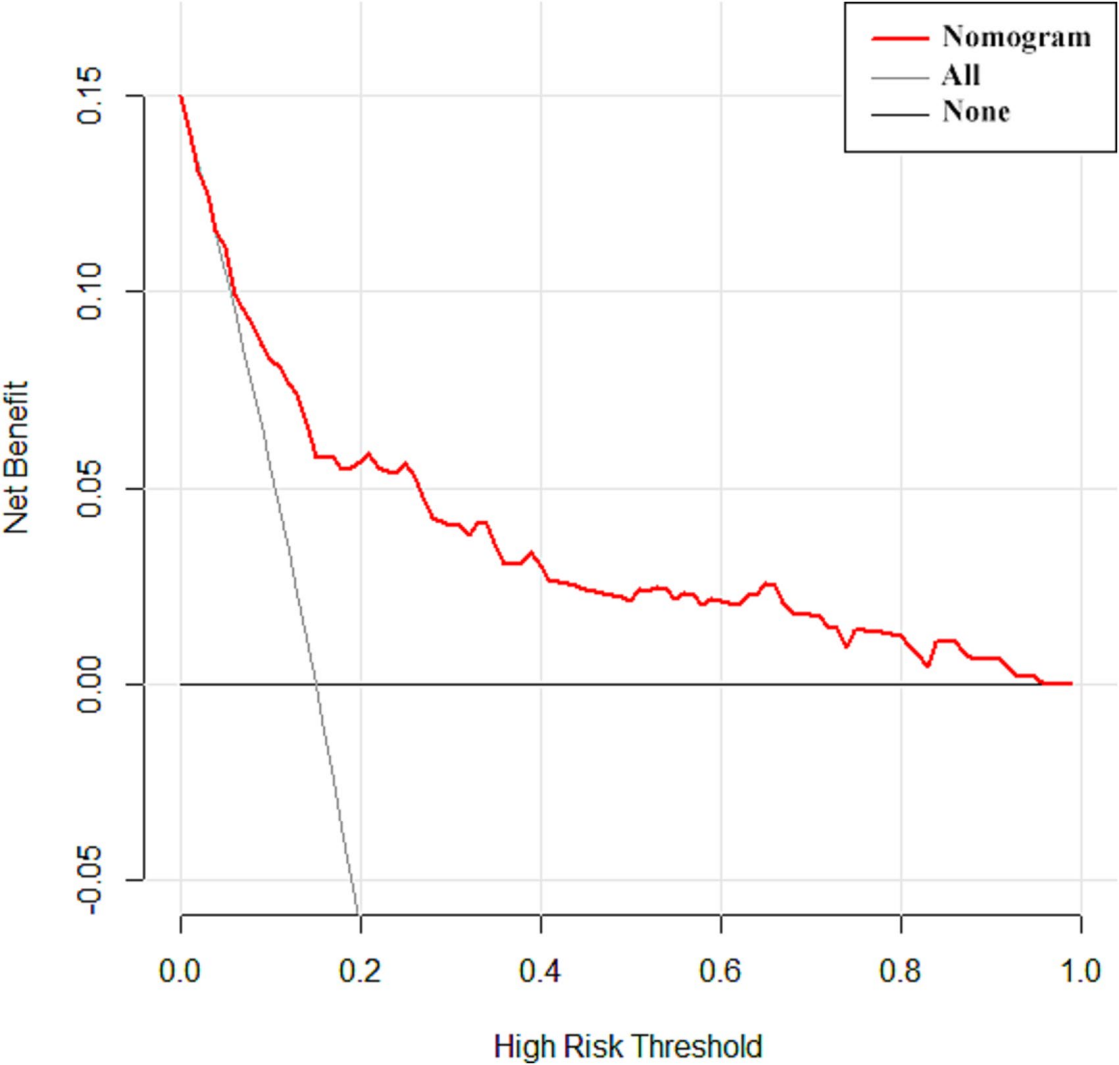
Clinical decision curve analysis of the fall risk prediction model for older adults with osteoporosis.

The ROC curve was drawn (Figure 2). The AUC was 0.798 (95% CI: 0.763–0.830), indicating good predictive capacity. The maximum Youden index was 0.485, with a cutoff value of 121.97 points, a sensitivity for fall prediction of 80.6%, and a specificity of 67.9%. Internal validation of the model yielded a C-index of 0.799 (95% CI: 0.798–0.801), indicating the model’s relatively good differentiation ability (Figure 3). The Hosmer-Lemeshow goodness-of-fit test showed χ^2^ = 8.147, *p* = 0.419, and a Brier score of 0.084, which is well below the threshold of 0.250, indicating good calibration. The calibration curve diagram (Figure 4) showed that the calibration and standard curves were almost overlapping, indicating that the predicted fall risk probabilities closely matched the observed probabilities. The DCA (Figure 5) showed that the clinical decision curve was consistently above the invalid lines, indicating the model’s potential clinical effectiveness when using interventions for individuals who have a fall risk probability in the range of 0.03 to 0.95.

## 4 Discussion

### 4.1 Analysis of fall occurrences in hospitalized older adults with OP

Within the 6-month follow-up period in this study, the incidence of falls among older adults with OP was 12.24%, and a subset of participants experienced multiple falls, with a multi-fall incidence rate of 2.07%. These findings are similar to the study results of Yu et al. (19). Among the 82 falls reported in this study, 60% were classified as injurious falls. The primary types of injuries included trauma (34%), soft tissue injuries (20%), and fractures (6%). These are similar to the findings of Yang et al. (20) who showed that bruises/contusions, fractures, and sprains/strains were the three most common types of fall injuries.

Regarding the time of occurrence, 85% of the falls occurred during the daytime, and this is consistent with results of other studies on older adults (21). Regarding the direct causes of falls, 31% of the falls were due to body discomfort experienced by the participants. Given the age-related decline in physical functions and various complications faced by older adults, those with OP exhibited decreased muscular strength (22), and reduced balance ability, significantly increasing their fall risk. Environmental factors accounted for 41% of falls, and these causes included obstacles on the ground and wet or uneven road surfaces, among others. This indicates a need for medical staff to educate older adults about recognizing and avoiding unsafe factors in their environment. A total of 12% of falls were due to high-risk activities of participants, such as riding a bicycle, rushing to beat traffic lights, and being distracted. Therefore, it is essential for older adults to prioritize their safety and avoid engaging in such high-risk activities in their daily lives that could result in falls, thereby safeguarding their own health and safety.

### 4.2 Analysis of fall risk factors in hospitalized older adults with OP

#### 4.2.1 Hospitalized older adults with OP who were females and had a fall history, fear of falling, and decreased balance ability were at greater risk of falls

The results of this study showed that female older adults had a significantly higher fall risk than male older adults, which is consistent with previous studies (23). Women tend to have lower peak bone mass than men and undergo rapid estrogen decline after menopause, which aggravates bone mass loss, thus resulting in more severe OP in older women compared to men (24). Therefore, for older women with OP, healthcare professionals should recommend appropriate estrogen supplementation (e.g., exogenous estrogen drugs) and advise incorporating estrogen-rich foods into their diet to balance endocrine levels, minimize the loss of bone mass, and delay the progression of OP.

We also found that older adults with OP who had a history of falls were at greater risk for future falls—a result consistent with results of earlier research (25). Such older adults typically exhibit a weaker constitution, have lower levels of activities of daily living, and significantly decreased lower limb muscle strength and walking stability, which severely impair postural control during walking. Furthermore, such individuals often experience a fear of falling (26).

Our results in this study also indicated that older adults with OP having FoF were more prone to falls. FoF can make older adults become overly focused on the possibility of a fall and deliberately avoid daily activities within the scope of their abilities. This avoidance limits mobility, reduces muscle strength, decreases balance ability, and ultimately increases fall risk. Medical professionals should assess fall history and causes for falls in such individuals, evaluate their awareness of fall risks, and educate them about recognizing possible fall risk factors. In addition, psychological evaluation of the extent of their FoF and management are crucial. For older adults with excessive FoF, healthcare providers should help develop a correct understanding of falls, reduce their sense of fear, and implement behavioral interventions to improve their levels of functioning and reduce fall risk.

It was also confirmed in this study that older adults with OP having decreased balance ability were at greater risk of falls, consistent with prior research findings (27). Aging leads to degeneration in various systems, including the nervous, sensory, and motor systems, which manifests as impaired limb function, delayed responses to external stimuli, weakened muscle strength, and decreasing balance ability over time (28). Besides, older adults with OP often had concurrent conditions such as low back pain and scoliosis, which further compromised balance and postural control. Enhancing balance through training has been shown to have a significant effect in improving postural control, enhancing muscle strength, strengthening stability, and optimizing balance ability among older adults, and thereby reducing their fall risk (29). Therefore, it is recommended that the healthcare professionals design systematic, standardized, and individualized balance rehabilitation training regimens for older adults with OP to enhance their postural control and muscle strength, improve balance stability, and reduce the likelihood of falls.

#### 4.2.2 Elevated serum calcium and BMD levels, as well as the use of walking aids, could significantly decrease fall risk in hospitalized older adults with OP

Our findings in this study revealed that serum calcium levels among older adults in the Non-fall group were significantly higher than those in the Fall group, suggesting that higher serum calcium levels are associated with a lower fall risk. Calcium plays a critical role in bone structure, and insufficient serum calcium can induce muscle spasms in limbs and trigger sensory impairments in older adults, which in turn increase neuromuscular excitability. In severe cases, this deficiency can lead to hand and foot spasms, heightening the risk of falls (30). Older adults with OP often have impaired vitamin D hydroxylation, which limits calcium retention in bones, contributing to reduced bone mass. Resistance to vitamin D impairs calcium absorption, further aggravating serum calcium deficiency and thus resulting in weakening bone density, which can induce or aggravate OP (31). To minimize these risks, healthcare providers should educate older adults about the importance of calcium and vitamin D supplementation to ensure adequate intake. It is recommended that postmenopausal older women maintain a calcium intake of up to 1,000 mg/day with regular monitoring to ensure that serum 25(OH)D levels are maintained at over 20 ng/mL (32). This regimen can help protect bone health and improve bone density, thus enhancing muscle strength and balance ability among older adults, ultimately reducing the risk of falls and fractures.

Additionally, we found that higher BMD levels correlated with lower fall risk in older adults with OP. Previous research has demonstrated the association between BMD and fall risk in older adults with OP, wherein the lower the BMD, the greater the loss of bone mass, and the higher the fall risk (33). Older adults with low BMD are more prone to vertebral compression fractures, which can lead to postural deformities such as a hunched back, further reducing decreased balance and stability. In addition, due to increased bone brittleness, such older adults are at a greater risk of spinal fracture even without significant trauma, leading to chronic pain in the chest and lower back, adversely affecting walking stability and increasing their fall risk. Regular monitoring of BMD for older adults is recommended to assess bone mass loss and the extent of OP. Early-stage anti-osteoporosis interventions should be implemented to avoid further BMD reduction to minimize fall risk and avoid complications such as OPF and other serious consequences.

Another notable finding was that the use of walking aids served as a protective factor against falls in older adults with OP, consistent with the results of Cohen et al. (34). In China, there is a lack of comprehensive evaluations for older adults in both clinical and community settings, leading many older adults to underestimate their functional decline and failing to adopt effective intervening measures in a timely manner. Walking aids can improve mobility, reduce fall risk, and lower the incidence of fall-related injuries, especially among older adults with concurrent balance and motor disorders. Therefore, healthcare providers should evaluate the need for walking aids among older adults with OP, ensure proper use of these aids, and offer guidance on selecting and using the most appropriate walking aid to optimize fall prevention strategies.

### 4.3 The prediction model of fall risk in hospitalized older adults with OP had relatively good predictive efficiency

Given the numerous fall risk factors in older adults with OP and the associated high incidence of falls, we used logistic regression in this study to analyze fall risk factors and constructed a nomogram. This model was found to perform well in terms of its predictive ability and clinical effectiveness, with a relatively high AUC of 0.898 (95% CI: 0.763–0.830).

Following internal validation, the model achieved a C-index value of 0.799 (95% CI: 0.798–0.801), reflecting a strong differentiation degree. The model’s sensitivity (80.6%) exceeded its specificity (67.9%), showing that it had a stronger ability to correctly identify older adults with high fall risk than to exclude those at low risk. This feature is especially advantageous for clinical settings, as it allows healthcare providers to identify high-risk individuals more accurately, facilitating more targeted fall prevention strategies.

The goodness of fit of the model was confirmed by the Hosmer-Lemeshow test, which yielded a *χ* of 8.147 and a *p* value of 0.419, indicating a good agreement between the model’s predicted fall risk and actual outcomes. Additionally, the calibration curve closely followed the reference line, indicating that the model’s predicted fall rates closely aligned with the actual observed fall rates, demonstrating its relatively good calibration ability. The Brier score for internal verification was 0.084, supporting the relatively good overall performance of the model, combining good differentiation and calibration.

From a clinical perspective, the model proved effective and within a wide range of predicted risk levels (3–95%), offering considerable benefits in interventions. By visualizing the model as a nomogram, medical staff can dynamically assess fall risk in older adults with OP and implement targeted preventive measures based on the presence of specific risk factors. This approach can help reduce the incidence of falls and improve their quality of life, underscoring the model’s practical utility in managing fall risk.

## 5 Conclusion

The fall risk prediction model for hospitalized older adults with OP developed in this study demonstrated strong differentiation, calibration, and clinical efficacy, making it a valuable tool for clinical personnel to estimate the probability of falls in this population and implement targeted preventive measures based on the presence of specific risk factors.

However, our study had the following limitations: This was a single-center study, with all participants selected from among patients hospitalized in the same grade 3A hospital, which introduces a certain degree of sampling bias. Moreover, external validation of the model’s predictive ability was not conducted, limiting its generalizability. Future research should focus on multi-center and large-sample studies for external validation and further refinement of the model to ensure its wider applicability and effectiveness in predicting fall risk among hospitalized older adults with OP.

## Data Availability

The original contributions presented in the study are included in the article/supplementary material, further inquiries can be directed to the corresponding author.

## Footnotes

^1^ http://www.nhc.gov.cn/wjw/lnjk/202211/89cb032e5a4a4b5499dfa9f0d23243ff.shtml

## References

[1] Muñoz M Robinson K Shibli-Rahhal A. Bone health and osteoporosis prevention and treatment. Clin Obstet Gynecol. (2020) 63:770–87. doi: 10.1097/GRF.0000000000000572, PMID: 33017332

[2] Tian L Luo C Li YF Wang QY Qu XL Yue C. Economic evaluation of four treatment strategies for postmenopausal patients with osteoporosis and a recent fracture in mainland China: a cost-effectiveness analysis. Arch Osteoporos. (2023) 18:100. doi: 10.1007/s11657-023-01309-8, PMID: 37460858

[3] Li H Jiang H Wang J Zhou J Liang H Chen G. Effects of mind-body exercises for osteoporosis in older adults: a systematic review and Meta-analysis of randomized controlled trials. Geriatr Orthop Surg Rehabil. (2023) 14:21514593231195237. doi: 10.1177/21514593231195237, PMID: 37588426 PMC10426313

[4] Lo JC Yang W Park-Sigal JJ Ott SM. Osteoporosis and fracture risk among older US Asian adults. Curr Osteoporos Rep. (2023) 21:592–608. doi: 10.1007/s11914-023-00805-7, PMID: 37542683 PMC10858302

[5] Barron RL Oster G Grauer A Crittenden DB Weycker D. Determinants of imminent fracture risk in postmenopausal women with osteoporosis. Osteoporos Int. (2020) 31:2103–11. doi: 10.1007/s00198-020-05294-3, PMID: 32613410 PMC7560920

[6] Qiu ML Xie Y Wang XH Wang XQ Zhao DB Zhou HQ. Practice guideline for patients with osteoporosis. Zhonghua Nei Ke Za Zhi. (2020) 59:953–9. doi: 10.3760/cma.j.cn112138-20200904-00792, PMID: 33256336

[7] Consensus on the application of hearing health assessment techniques for Chinese older adult (draft). Chinese J Geriatric Care. (2019) 17:37–9.

[8] Collins GS Reitsma JB Altman DG Moons KGM. Transparent reporting of a multivariable prediction model for individual prognosis or diagnosis (TRIPOD): the TRIPOD statement. BMJ. (2015) 350:g7594. doi: 10.1136/bmj.g7594, PMID: 25569120

[9] Lumini A Nanni L. Convolutional neural networks for ATC classification. Curr Pharm Des. (2018) 24:4007–12. doi: 10.2174/1381612824666181112113438, PMID: 30417778

[10] Ortega-Bastidas P Gómez B Aqueveque P Luarte-Martínez S Cano-de-la-Cuerda R. Instrumented timed up and go test (iTUG)-more than assessing time to predict falls: a systematic review. Sensors. (2023) 23:3426. doi: 10.3390/s23073426, PMID: 37050485 PMC10098780

[11] Chen LK Woo J Assantachai P Auyeung TW Chou MY Iijima K. Asian working Group for Sarcopenia: 2019 consensus Update on sarcopenia diagnosis and treatment. J Am Med Dir Assoc. (2020) 21:300–307.e2. doi: 10.1016/j.jamda.2019.12.012, PMID: 32033882

[12] Muñoz-Bermejo L Adsuar JC Mendoza-Muñoz M Barrios-Fernández S Garcia-Gordillo MA Pérez-Gómez J. Test-retest reliability of five times sit to stand test (FTSST) in adults: a systematic review and Meta-analysis. Biology. (2021) 10:510. doi: 10.3390/biology10060510, PMID: 34207604 PMC8228261

[13] Baek JY Jung HW Kim KM Kim M Park CY Lee KP. Korean working group on sarcopenia guideline: expert consensus on sarcopenia screening and diagnosis by the Korean Society of Sarcopenia, the Korean Society for Bone and Mineral Research, and the Korean geriatrics society. Ann Geriatr Med Res. (2023) 27:9–21. doi: 10.4235/agmr.23.0009, PMID: 36958807 PMC10073972

[14] Lima CA Ricci NA Nogueira EC Perracini MR. The berg balance scale as a clinical screening tool to predict fall risk in older adults: a systematic review. Physiotherapy. (2018) 104:383–94. doi: 10.1016/j.physio.2018.02.002, PMID: 29945726

[15] Buckley RA Atkins KJ Fortunato E Silbert B Scott DA Evered L. A novel digital clock drawing test as a screening tool for perioperative neurocognitive disorders: a feasibility study. Acta Anaesthesiol Scand. (2021) 65:473–80. doi: 10.1111/aas.13756, PMID: 33296501

[16] Snellman S Hörnsten C Olofsson B Gustafson Y Lövheim H Niklasson J. Validity and test-retest reliability of the Swedish version of the geriatric depression scale among very old adults. BMC Geriatr. (2024) 24:261. doi: 10.1186/s12877-024-04869-7, PMID: 38500031 PMC10946128

[17] Chinese expert consensus on exercise prescription (2023). Chinese J Sports Med. (2023) 42:3–13. doi: 10.16038/j.1000-6710.2023.01.012

[18] Buysse DJ Reynolds CF 3rd Monk TH Berman SR Kupfer DJ. The Pittsburgh sleep quality index: a new instrument for psychiatric practice and research. Psychiatry Res. (1989) 28:193–213. doi: 10.1016/0165-1781(89)90047-4, PMID: 2748771

[19] Jiang Y Sun YJ Zhou P. A study of the relationship between osteoporosis and falls in community-dwelling older adults. Chinese J Preven Control Chronic Dis. (2018) 26:102–6. doi: 10.16386/j.cjpccd.issn.1004-6194.2018.02.006

[20] Yang YC Lin MH Wang CS Lu FH Wu JS Cheng HP. Geriatric syndromes and quality of life in older adults with diabetes. Geriatr Gerontol Int. (2019) 19:518–24. doi: 10.1111/ggi.13654, PMID: 30957935

[21] Zhang L Zeng Y Weng C Yan J Fang Y. Epidemiological characteristics and factors influencing falls among elderly adults in long-term care facilities in Xiamen, China. Medicine. (2019) 98:e14375. doi: 10.1097/MD.0000000000014375, PMID: 30813138 PMC6407997

[22] Simon A Schäfer HS Schmidt FN Stürznickel J Amling M Rolvien T. Compartment-specific effects of muscle strength on bone microarchitecture in women at high risk of osteoporosis. J Cachexia Sarcopenia Muscle. (2022) 13:2310–21. doi: 10.1002/jcsm.13044, PMID: 35852049 PMC9530535

[23] SUH M KIM D CHO I HAM O. Age and gender differences in fall-related factors affecting community-dwelling older adults. J Nurs Res. (2023) 31:e270:e270. doi: 10.1097/jnr.0000000000000545, PMID: 36863032

[24] Patsch JM Deutschmann J Pietschmann P. Gender aspects of osteoporosis and bone strength. Wien Med Wochenschr. (2011) 161:117–23. doi: 10.1007/s10354-011-0891-9, PMID: 21461801

[25] Arnold CM Busch AJ Schachter CL Harrison L Olszynski W. The relationship of intrinsic fall risk factors to a recent history of falling in older women with osteoporosis. J Orthop Sports Phys Ther. (2005) 35:452–60. doi: 10.2519/jospt.2005.35.7.452, PMID: 16108586

[26] Stanghelle B Bentzen H Giangregorio L Pripp AH Skelton DA Bergland A. Effects of a resistance and balance exercise programme on physical fitness, health-related quality of life and fear of falling in older women with osteoporosis and vertebral fracture: a randomized controlled trial. Osteoporos Int. (2020) 31:1069–78. doi: 10.1007/s00198-019-05256-4, PMID: 31925473

[27] Ferrara PE Salini S Maggi L Foti C Maccauro G Ronconi G. Evaluation of quality of life and static balance in postmenopausal osteoporosis women after tai chi Chuan practice: an observational randomized case control study. J Biol Regul Homeost Agents. (2019) 33:163–9. PMID: 31172734

[28] Dai XH Lu YY Zhegn ZY. Effects of Baduanjin and fitness walking on stability and muscle strength of middle-aged and old women. Chinese J Rehabil Med. (2023) 38:319–24.

[29] Wang RX Peng N Niu XR. Intensive balance training lowers the incidence of falls of poststroke patient. Chinese Prev Med. (2019) 20:128–31. doi: 10.16506/j.1009-6639.2019.02.010

[30] Tan L He R Zheng X. Effect of vitamin D, calcium, or combined supplementation on fall prevention: a systematic review and updated network meta-analysis. BMC Geriatr. (2024) 24:390. doi: 10.1186/s12877-024-05009-x, PMID: 38698349 PMC11064304

[31] Tang G Feng L Pei Y Gu Z Chen T Feng Z. Low BMI, blood calcium and vitamin D, kyphosis time, and outdoor activity time are independent risk factors for osteoporosis in postmenopausal women. Front Endocrinol. (2023) 14:1154927. doi: 10.3389/fendo.2023.1154927, PMID: 37937050 PMC10627178

[32] Camacho PM Petak SM Binkley N Diab DL Eldeiry LS Farooki A. American association of clinical endocrinologists/AMERICAN COLLEGE of endocrinology clinical practice GUIDELINES for the diagnosis and treatment of postmenopausal OSTEOPOROSIS-2020 UPDATE. Endocr Pract. (2020) 26:1–46. doi: 10.4158/GL-2020-0524SUPPL, PMID: 32427503

[33] Ma C Liu A Sun M Zhu H Wu H. Effect of whole-body vibration on reduction of bone loss and fall prevention in postmenopausal women: a meta-analysis and systematic review. J Orthop Surg Res. (2016) 11:24. doi: 10.1186/s13018-016-0357-2, PMID: 26888467 PMC4758089

[34] Cohen D Morrison A. Interventions for preventing falls among older adults living in the community. Am Fam Physician. (2017) 95:152–3. PMID: 28145670

